# Individual and community-level factors associated with adequate antenatal care service utilization in sub-Saharan Africa

**DOI:** 10.1186/s41182-024-00631-2

**Published:** 2024-10-15

**Authors:** Setegn Muche Fenta, Haile Mekonnen Fenta, Seyifemickael Amare Yilema, Ding-Geng Chen, Amsalu Worku Mekonnin

**Affiliations:** 1 Department of Statistics, College of Natural and Computational Sciences, Debre Tabor University, Debre Tabor, Ethiopia; 2 Department of Statistics, College of Science, Bahir Dar University, Bahir Dar, Ethiopia; 3 Department of Statistics, University of Pretoria, Pretoria, South Africa; 4 Department of Obstetric and Gynecology, College of Medicine and Health Sciences, Bahir Dar University, Bahir Dar, Ethiopia

**Keywords:** Antenatal care, Sub-Saharan Africa, Demographic and health survey, Hierarchical models

## Abstract

**Background:**

Sub-Saharan Africa (sSA) continues to rank among the regions in the world with the highest rates of maternal mortality and the lowest rates of utilization of maternal health care. The risk of death for women in sSA is 268 times higher than that of women in high-income nations. Adequate antenatal care (ANC) services utilization is essential to the mother's and the baby's survival and well-being. This study aimed to identify both individual and community-level factors associated with adequate antenatal care services utilization in sSA.

**Method:**

We used data from the most recent Health and Demographic Surveys (DHS), which were carried out between 2012 and 2022 in 33 sSA countries. A total of 240,792 women were included in this study. The two-level mixed-effects logistic regression model was used to identify the individual and community-level factors associated with the use of adequate ANC service.

**Results:**

The pooled prevalence of adequate ANC service utilization in sSA was 55.48% (95% CI: 55.28–55.68). The study showed that secondary and above-educated women (AOR = 2.13, 95% CI 2.07–2.19, secondary and above-educated husbands (AOR = 1.55, 95% CI 1.51–1.60), rich women AOR = 1.26, 95% CI 1.24–1.29), women 35–49 years of age (AOR = 1.36, 95% CI 1.32–1.41) and distance to a health facility is not a big problem (AOR = 1.13; 95% CI 1.11–1.16) was significantly and positively correlated with the use of adequate ANC services. However, rural women (AOR = 0.80; 95% CI 0.78–0.82), not having mass media access (AOR = 0.74, 95% CI 0.72–0.75), 5 and above birth order (AOR = 0.73, 95% CI 0.68–0.78) were significantly and negatively correlated with the use of adequate ANC services. Additionally, the random effects model showed that variables at the community and individual levels were responsible for approximately 62.60% of the variation in the use of adequate ANC services.

**Conclusion:**

The sSA countries had a low prevalence of adequate utilization of ANC with a significant variation among countries. Moreover, public health initiatives should focus on rural women, poor women, and uneducated women to enhance maternal health services utilization. Furthermore, policies and programs that address cluster variations in the utilization of adequate ANC services must be developed, and their implementation must be vigorously pursued.

## Introduction

In recent decades, maternal and child deaths have become a global health priority. However, the risk of these problems is even higher for developing countries. The third Sustainable Development Goal (SDG) focuses on reducing maternal mortality and improving women's health care: aiming to reduce the global maternal mortality ratio (MMR) to 70 per 100,000 live births by 2030 [1, 2]. Despite progress and efforts, nearly 800 women die each day as a result of avoidable pregnancy and childbirth complications. In 2020, approximately 287,000 women died as a result of pregnancy and birth. Nearly 95% of maternal deaths in 2020 occurred in low- and lower-middle-income countries. The sSA and Southern Asia accounted for approximately 87% (253,000) of global maternal deaths in 2020. The sSA and Southern Asia were responsible for approximately 87% (253,000) of the estimated global maternal deaths in 2020. sSA alone was responsible for 70% (202,000) of maternal deaths, with Southern Asia accounting for 16% (47,000) [3–5]. Women and newborns can be saved if they receive skilled medical care before, during, and after childbirth. Antenatal care (ANC) visits are recommended by the World Health Organization (WHO) as a key strategy for improving the health of pregnant women. The organization previously recommended at least four ANC visits during the pregnancy. However, it changed its recommended minimum number of ANC visits from four to eight contacts to ensure a safe pregnancy and healthy baby [6, 7]. Approximately 90% of women worldwide have received skilled ANC services at least once, with only 60% utilizing skilled ANC services at least four times. sSA has the lowest rates of antenatal care, with only 49% of women receiving at least four skilled ANC services[8, 9].

Earlier studies conducted in various sSA countries to identify factors associated with adequate ANC services utilization have been institutional-based [10–12] and considered only individual factors [12–15]. Most of the research mentioned above was carried out in a single country [13, 14, 16]. However, community-level factors like residence [17, 18], region [17, 18], media exposure [14, 17], distance to health center [17, 18], and cluster (enumeration areas) [17, 19] may have an impact on the utilization of adequate ANC services. Besides, the above studies did not employ a multi-country approach to identify factors associated with adequate ANC services based on the recent pooled DHS data. The previously mentioned study also used an ordinary logistic regression model to identify the factors associated with the use of adequate ANC services [10–12].

An ordinary logistic regression model assumes that each woman is independent and cannot take into account the hierarchical structure of the data within communities. The effect would be an underestimation of the standard errors for the covariate effects due to the strong homogeneity among women in communities. This could result in incomplete and misleading conclusions regarding the relationship between the explanatory variable of the use of adequate ANC services and the use of adequate ANC services [20, 21]. Multilevel logistic regression models are appropriate for statistical analysis of binary outcome data that are hierarchical or clustered [22]. Multilevel data analysis is primarily done for two reasons: first, to ensure accurate inferences by taking into account potential variation between level-2 units, like communities or clusters; second, to evaluate the contribution of level-2 determinant factors and unexplained variation between level-2 units (clusters) to the explanation of variations in the proneness of individuals (level-1 units) to the binary outcome [20, 23, 24]. To investigate whether cluster-level covariates and the unexplained between-cluster heterogeneity account for the individual binary outcomes, a number of multilevel logistic regression techniques have been proposed. The unobserved heterogeneity between clusters in multilevel models is typically measured using the intra-class correlation coefficient (ICC), proportional change in variance (PCV), and median odds ratio (MOR) due to their ease of interpretation, particularly in linear mixed models [24–26]. Accordingly, the current study uses a multilevel mixed effect analysis of the most recent DHS data to examine the individual and community-level factors associated with the utilization of adequate ANC care services in sSA countries. Furthermore, the results of this study encourage health planners, policymakers, sponsors, and health professionals in their efforts to further enhance pregnant women's use of adequate ANC services, which will help reduce maternal mortality in sSA. Therefore, this study aimed to identify both individual and community-level factors associated with adequate antenatal care services utilization in sSA.

## Method

### Data source

**Fig. 1:**
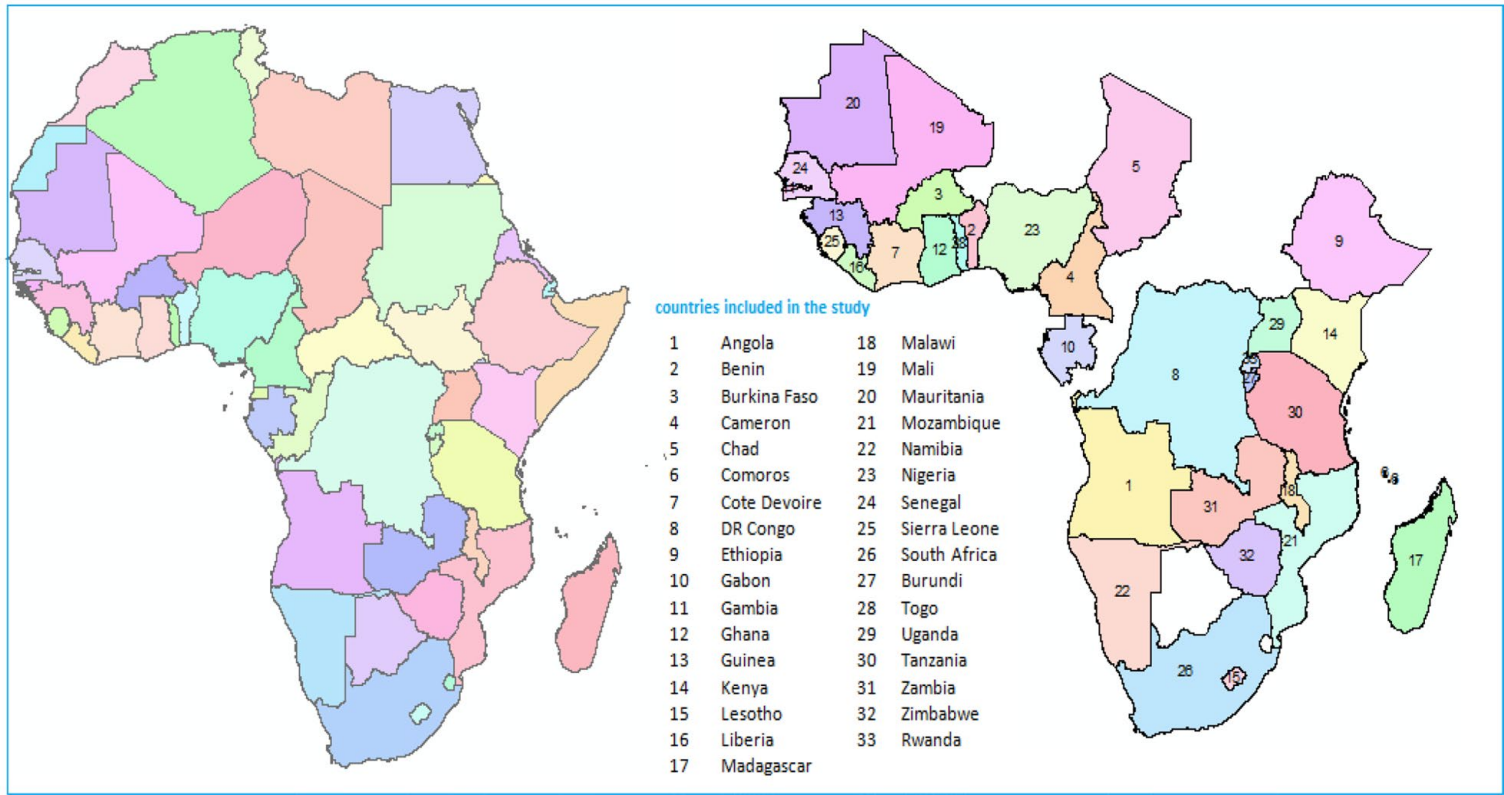
Sampling procedures to select the eligible countries and children for the study

This study used data from the most recent DHS in sSA countries from 2012 to 2022. The data were collected from 33 sSA countries (Fig. 1). A total of 240,192 women participated in this study. The DHS data were combined to analyze the prevalence and factors influencing ANC service utilization in sSA. The DHS is a nationwide survey that collects data on basic health indicators such as mortality, morbidity, family planning, fertility, and maternal/child health. The data came from the DHS program. Each country's survey consists of various datasets, including men, women, children, birth, and household datasets. For this study, a birth record (BR) file was used. Figure 1 depicts the geographical area of this study. The number in Fig. 1 represents the 33 sSA countries' geographical location. Table 1 shows the survey year and the total number of respondents for 33 sSA countries (Fig. 1 and Table 1).

**Table 1:** Pooled demographic and health surveys (DHS) data from 33 sSA countries, 2012–2022

Country	Year of survey	Weighted sample (n)	Weighted sample (%)
Central region of Africa		48,727	
Angola	2017/18	8,947	3.7
Cameroon	2018	6,463	2.7
Congo DR	2013/14	11,293	4.7
Congo	2011/12	6,463	2.7
Chad	2014/15	11,104	4.6
Gabon	2019/21	4,457	1.9
Western region of Africa		82,762	
Burkina Faso	2021	6,428	2.7
Benin	2017/18	8,994	3.7
Cote d’Ivoire	2021	5,670	2.4
Gambia	2019/20	5,799	2.4
Ghana	2022	5,218	2.2
Guinea	2018	5,530	2.3
Liberia	2019/20	4,267	1.8
Mali	2018	6,368	2.6
Niger	2012	7,680	3.2
Nigeria	2018	21,792	9.1
Togo	2013/14	5,016	2.1
Eastern region of Arica		99,697	
Burundi	2016/17	8,660	3.6
Comoros	2012	2,016	.8
Ethiopia	2016	7,193	3.0
Kenya	2022	10,412	4.3
Madagascar	2021	9,315	3.9
Malawi	2015/16	13,448	5.6
Mauritania	2019/21	7,640	3.2
Mozambique	2015	4,014	1.7
Namibia	2013	3,974	1.7
Rwanda	2019	6,167	2.6
Tanzania	2022	5,825	2.4
Uganda	2016	10,263	4.3
Zambia	2018	7,372	3.1
Zimbabwe	2015	7,372	3.1
Southern region of Africa		9,606	
Lesotho	2014/15	2,596	1.1
South Africa	2016	3,036	1.3
Total		240,792	100.0

### Outcome variable

Adequate ANC visits were used as the outcome variable of the study. According to the WHO definition [27], adequate ANC services utilization was defined as women receiving four and above ANC services during pregnancy is considered adequate.

### Independent variables

**Fig. 2:**
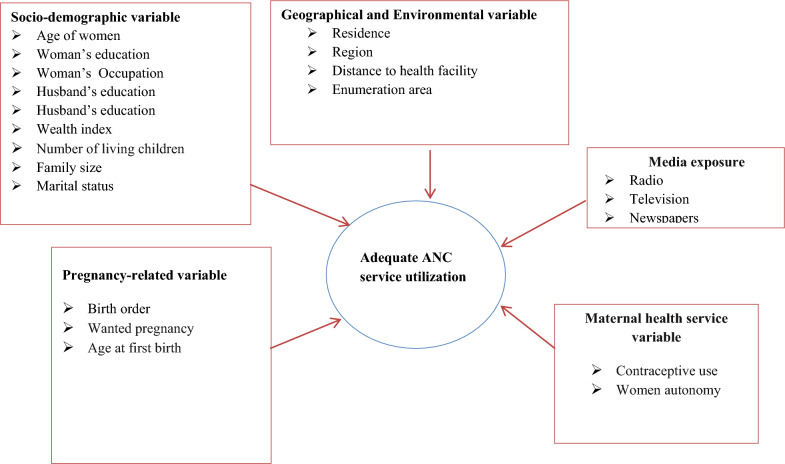
Conceptual framework for the relationship between adequate ANC services utilization and variables in sSA from 2012 to 2022

The explanatory variables for adequate ANC service utilization were classified as individual and community level. These variables were chosen based on various works of literature [13, 17, 28, 29]. Individual-level variables include mothers' ages, wealth indexes, employment statuses, maternal education levels, husband’s education levels, number of living children, family size, age at first birth, women's decision-making capacity, wanted pregnancy, contraceptive use, and birth order. In addition, residence, distance to the health facility, exposure to mass media, cluster (enumeration area), and region were considered community-level variables (Fig. 2).

### Statistical analysis

To make relevant inferences, the data were weighted using sampling weight (v005), primary sampling unit (v023), and strata (v021). The DHS surveys provide dependent data, which is organized in a hierarchical or multilevel structure due to the nesting of women within enumeration areas or clusters. Women who reside in the same clusters are likely to experience similar outcomes because they have comparable characteristics and access to the same maternal health services. Accordingly, women who are randomly selected from two randomly selected households within the same cluster or enumeration area may have a similar status of adequate ANC service utilization than women who are randomly selected from two different clusters, even in cases where the measured household or individual-specific characteristics of the selected households are the same.

The adequate ANC services utilization data have been analyzed using the standard binary logistic regression model. However, this model ignores potential intra-cluster correlation among observations within a cluster and may produce biased results and conclusions because it assumes that the variable conditional on which the individual women's utilization of adequate ANC services is independent [30, 31]. Furthermore, this model does not take into account the hierarchical structure of the data and does not allow for the examination of the correlation between specific cluster characteristics and individual outcomes [32]. To solve this problem, we applied a multilevel logistic regression model. The multilevel logistic regression model divides the total individual variance into variation attributed to the clusters and the remaining individual-level variation. This allows cluster-specific random effects, which account for the dependency of the observations. The multilevel logistic regression model can accurately estimate standard errors, produce more accurate inferential decisions, and enable the investigation of sources of variations both within and between clusters because they account for the clustered nature of the data [27]. The multilevel logistic regression model with random effects for outcome ${y}_{ij}$ is expressed as follows:

$$log\left(\frac{{\pi }_{ij}}{1-{\pi }_{ij}}\right)=g\left({\mu }_{ij}\right)={{\varvec{X}}}_{ij}^{T}{\varvec{\beta}}+{{\varvec{Z}}}_{j}^{T}\boldsymbol{\alpha }+{u}_{j},$$

where $g(.)$ is the link function, $i$ and $j$ are the level 1 (individual women) and level 2 (community) units, respectively; ${{\varvec{X}}}_{ij}=\left(1,{x}_{1ij}, . . . , {x}_{pij}\right)$ refer to a vector of $p$ covariates measured on the $i$ individual and ${{\varvec{Z}}}_{j}=\left(1,{z}_{1j}, . . . , {z}_{qj}\right)$ is a vector of $q$ covariates measured on the $j$ community-level variables, ${\varvec{\beta}}$ and $\boldsymbol{\alpha }$ are a vector of fixed regression parameters, and ${\pi }_{ij}$ is the likelihood of receiving adequate ANC service for the ${i}^{th}$ women in the ${j}^{th}$ community, ${u}_{j}$ showed the random effect assumed independently and normally distributed with mean zero and variance${\sigma }_{u}^{2}$, in short${u}_{j}\sim N\left(0,{\sigma }_{u}^{2}\right)$. In multilevel binary logistic regression, four models are considered: model I (only individual-level variables), model II (only community-level variables), model III (both individual and community-level variables), and the null model. The null model was used to examine the variability of adequate ANC visits across the enumeration area. It was determined which community-level variables and which individual-level variables were associated with the outcome variables (Model II and Model I, respectively). The final model (Model III) included both individual and community-level variables, as well as the outcome variable (adequate ANC visits). The fixed effect is reported in the form of an adjusted odds ratio with a 95% confidence interval. Statistical significance was determined for all variables with p-values < 0.05. Random effects or measures of variation, such as the intra-cluster correlation coefficient (ICC), median odds ratio (MOR), and proportional change in variance (PCV), were used to assess the variation in adequate ANC visits across clusters. The ICC quantifies the degree of heterogeneity of adequate ANC visits between clusters. It was calculated using the following formula:$ICC= \frac{{V}_{A}}{{V}_{A}+{\pi }^{2}/3}= \frac{{V}_{A}}{{V}_{A}+3.29}$, where ${V}_{A}$ is the estimated variance in each model. The ICC has a range of 0 to 1. The observed results are completely independent if the ICC value is 0, and completely dependent on the cluster to which they belong if the value is 1. The correlations are low, moderate, high, and very high, respectively, according to the ICC values of less than 0.50, between 0.50 and 0.75, between 0.76 and 0.90, and greater than 0.90 [33]. The PCV shows the variation in adequate ANC visits explained by determinants and computed as $\text{PCV}= \frac{{V}_{A}-{V}_{B}}{{V}_{A}}$, where ${V}_{A}$ = variance of the initial model, and ${V}_{B}$ = variance of the model with more terms [33]. The MOR is the median value of the odds ratio, which quantifies the variation in adequate ANC visits between clusters in terms of the odds ratio scale and is defined as the median value of the odds ratio between the clusters at a high likelihood of adequate ANC visits and the cluster at lower risk when randomly selecting individuals from two clusters. It was computed using the formula:$MOR=\text{exp}(\sqrt{2*{V}_{A}*0.6745} )\approx \text{exp}(0.95\sqrt{{V}_{A}} )$, where ${V}_{A}$ is the cluster-level variance. The MOR measure is always greater than or equal to 1. If the $MOR$ is 1, there is no variation between clusters [33–36]. Multicollinearity was tested using the variance inflation factor (VIF) test, suggesting that there was no multicollinearity since all variables had VIF < 5 and a tolerance greater than 0.1. The candidate model was compared using the deviance information criterion (DIC), Akaike's information criterion (AIC), and Bayesian information criterion (BIC). The model that has the lowest information criterion value will be chosen as the analysis's best model [37].

## Result

### Socio-demographic characteristics of study participants

A total of 240,792 pregnant women were included in this study. Of the total, 108,075 (44.9%) were between the ages of 25 and 34. About 85,741 (35.6%) of the mothers did not attend formal education, while 144,122 (59.9%) of the women were working in any sector. The majority, 223,804(92.9%) of the pregnancies were wanted, while 16,988 (9%) of the pregnancies were unwanted. Almost half (46.0%) of women had a poor wealth index and 34.10% had a rich wealth index. The majority of women, 162,719 (67.6%) lived in rural areas, and15, 369 (71.7%) of mothers were married (Table 2).

**Table 2:** Socio-demographic characteristics of study participants

Variable	Frequency	Percentage
Age of women		
15–24	73,876	30.7
25–34	108,075	44.9
35–49	58,841	24.4
Woman’s education		
No education	85,741	35.6
Primary education	80,847	33.6
Secondary and above	74,204	30.8
Woman’s occupation		
Housewives	96,670	40.1
Working any sector	144,122	59.9
Husband’s education		
No education	112,869	46.9
Primary education	54,734	22.7
Secondary and above	73,189	30.4
Husband’s occupation		
Not working	50,690	21.1
Working	190,102	78.9
Birth order		
1	51,096	21.2
2–4	114,189	47.4
5 and above	75,507	31.4
Number of living children		
1	56,591	23.5
2–3	88,840	36.9
4 and above	95,361	39.6
Sex of household head		
Male	187,050	77.7
Female	53,742	22.3%
Women’s decision-making capacity		
Women alone	34,959	14.5
Women and her husband	79,045	32.8
Husbands alone	126,788	52.7
Contraceptive use		
No	163,997	68.1
Yes	76,795	31.9
Wealth index		
Poor	110,748	46.0
Middle	47,944	19.9
Rich	82, 100	34.1
Residence		
Urban	78,073	32.4
Rural	162,719	67.6
Getting money needed for treatment		
Big problem	138,281	57.4
Not a big problem	102,511	42.6
Distance to a health facility		
Big problem	107,125	44.5
Not a big problem	133,667	55.5
Getting medical help for self: not wanting to go alone		
Big problem	70,636	29.3
Not a big problem	170,156	70.7
Access to mass media		
Yes	148,017	61.5
No	92,775	38.5
Age at 1st birth in years		
< 20 years	163,673	68.0
21–25 years	55,958	23.2
26 and above years	21,161	8.8
Family size		
1–3	33,710	14.0
4–6	106,000	44.0
7 and above	101,082	42.0
Pregnancy wanted		
Yes	223,804	92.9
No	16,988	7.1

### The pooled adequate prevalence of ANC service utilization in sSA

**Fig. 3:**
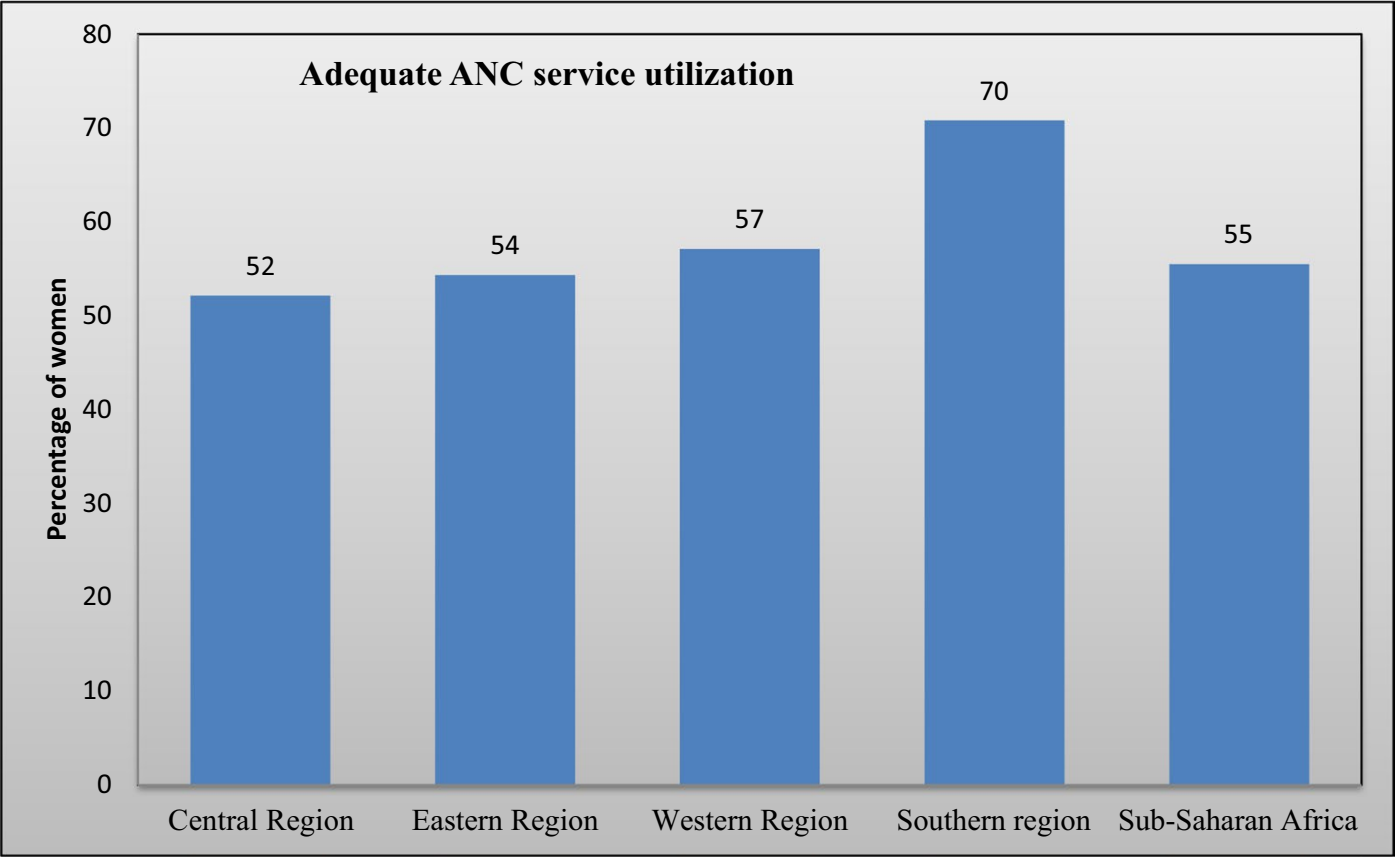
Prevalence of adequate ANC service utilization in sSA

The prevalence of using adequate ANC services in sSA is summarized in Fig. 3. The pooled prevalence of adequate ANC service utilization in sSA countries was 55% (95% CI: 54–56). The prevalence of adequate ANC service utilization varied significantly across the sSA region. The central sSA region (52%) had the lowest proportion of adequate ANC service utilization coverage, while the Southern sSA region (70%) had the highest. Furthermore, the prevalence of adequate ANC service utilization in the Western and Eastern sSA regions were 57% and 54%, respectively (Fig. 3).

### The prevalence of adequate ANC service utilization across sSA countries

**Fig. 4:**
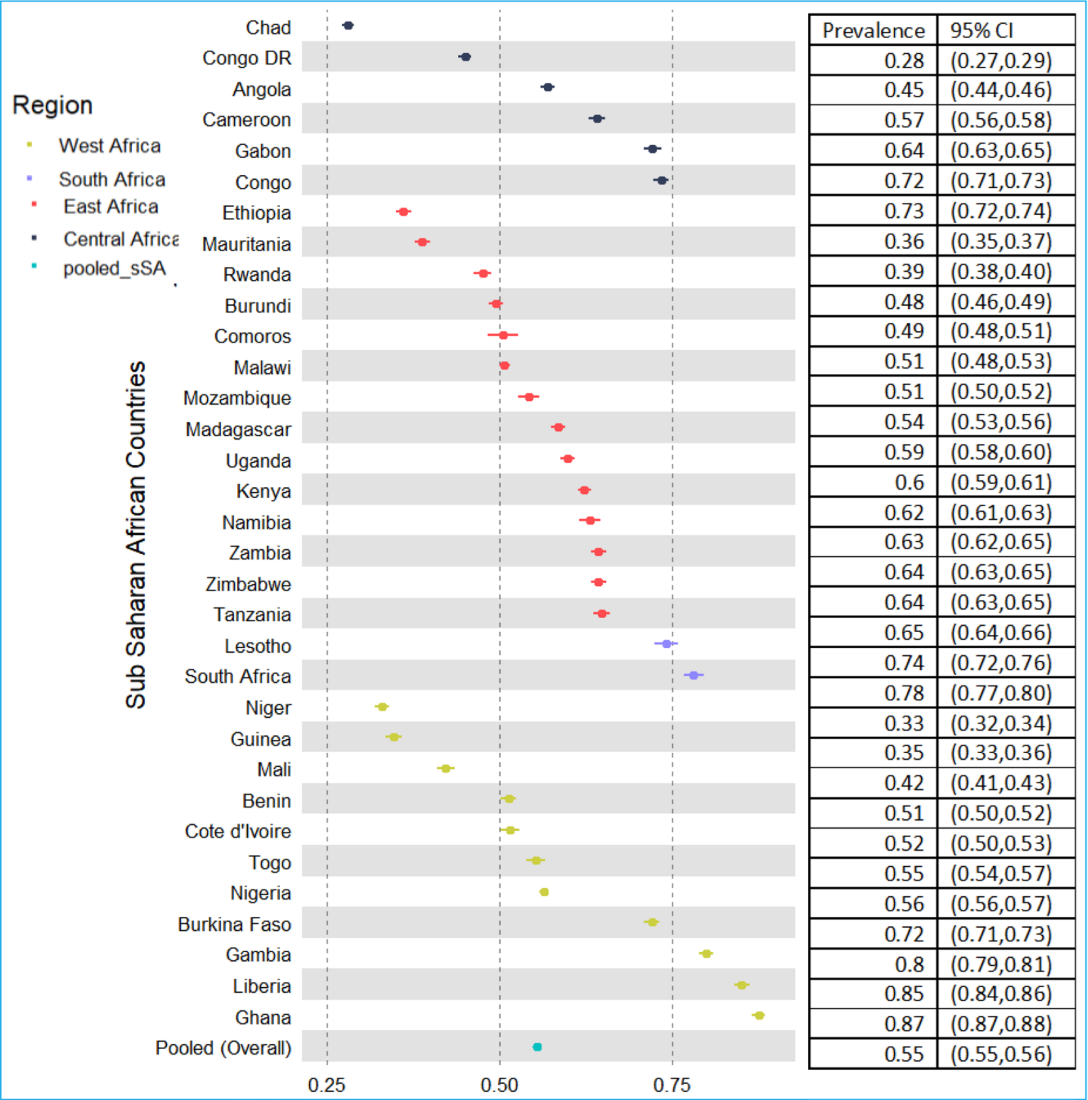
Forest plot of adequate ANC service utilization across sSA countries

The prevalence of adequate ANC service utilization across sSA countries is shown in Fig. 4. There were notable variations in the prevalence of adequate ANC service utilization among the countries in sSA. Among the sSA countries, Ghana (87%), Liberia (85%), Gambia (80), and South Africa (78%) have the highest rates of adequate ANC service utilization, while Niger (33%), Guinea (34%), Ethiopia (36%), and Mauritania (38) have the lowest rates (Fig. 4).

### Factors associated with the use of adequate ANC services visit utilization in sSA countries

The results of the two-level mixed effect logistic regression model are summarized in Table 3.

**Table 3:** Two-level mixed effect logistic regression analysis of factors associated with the use of adequate ANC services visit utilization in sSA countries

Variable	Model II AOR (95% CI)	Model III AOR (95% CI)	Model IV AOR (95% CI)
Age of women			
15–24	1		1
25–34	1.24(1.21,1.27)*		1.22(1.19, 1.25)*
35–49	1.39(1.35,1.44)*		1.36(1.32, 1.41)*
Woman’s education			
No education	1		1
Primary education	1.35(1.32, 1.38)*		1.51(1.47, 1.54)*
Secondary and above	2.05(1.99, 2.10)*		2.13(2.07, 2.19)*
Woman’s occupation			
Housewives	1		
Working any sector	1.18(1.15, 1.20)*		1.17(1.14, 1.17)*
Husband’s education			
No education	1		1
Primary education	1.15((1.12, 1.18)		1.27(1.24, 1.32)*
Secondary and above	1.51(1.47, 1.55)		1.55(1.51, 1.60)*
Wealth index			
Poor	1		1
Middle	1.05(1.03,1.17)*		1.10(1.08, 1.12)*
Rich	1.15(1.13,1.18)*		1.26(1.24,1.29)*
Age at 1st birth in years			
< 20 years	1		1
21–25 years	1.38(1.36, 1.41)*		1.27(1.25, 1.29)*
26 and above years	1.58(1.56, 1.61)*		1.52(1.46, 1.55)*
Pregnancy wanted			
Yes	1		1
No	0.80 (0.77, 0.82)*		0.81(0.78, 0.84)*
Getting money needed for treatment			
Big problem	1		1
Not a big problem	1.17(1.15,1.19)*		1.13(1.11, 1.16)*
Getting medical help for self: not wanting to go alone			
Big problem	1		1
Not a big problem	1.21(1.19, 1.24)*		1.09(1.07,1.12)*
Contraceptive use			
No	1		1
Yes	1.34((1.31,1.36)*		1.39(1.36,1.42)*
Husband’s occupation			
Not working	1		1
Working	0.88(0.86, 0.91)*		0.83(0.81, 0.86)*
Birth order			
First	1		1
2–4	0.85(0.80, 0.90)*		0.84(0.79, 0.89)*
5 and above	0.73(0.69, 0.78)*		0.73(0.68, 0.78)*
Number of living children			
1	1		1
2–3	0.99(0.94, 1.05)		1.01(.095, 1.07)
4 and above	0.96(0.90, 1.01)		0.98(0,92, 1.05)
Sex of household head			
Male	1		1
Female	1.09(1.06, 1.11)*		1.10(1.07, 1.12)*
Women’s decision-making capacity			
Women alone	1		1
Women and her husband	1.03(1.01, 1.06)*		1.01(1.98, 1.04)
Husbands alone	0.87(0.84, 0.90)*		0.83(0.81, 0.86)*
Family size			
1–3	1		1
4–6	0.99(0.96, 1.02)		0.99(0.97, 1.07)
7 and above	1.01(0.98, 1.04)		1.00(0.97, 1.03)
Marital status			
Married	1		1
Single	1.09(1.05,1.14)*		1.10(1.05, 1.15)*
Widowed/divorced	1.05(0.99, 1.12)*		1.10(1.04,1.16)*
Region			
Central region		1	1
Eastern region		1.04(1.02, 1.06)*	0.92(0.90, 0.95)*
Western region		1.05(1.03, 1.08)*	1.37(1.33, 1.40)*
Southern region		1.77(1.68, 1.85)*	1.22(1.16, 1.29)*
Residence			
Urban		1	1
Rural		0.64(0.62,0.65)	0.80(0.78, 0.82)*
Access to mass media			
Yes		1	1
No		0.56(0.55, 0.57)*	0.74(0.72, 0.75)*
Distance to a health facility			
Big problem		1	1
Not a big problem		1.38(1.35, 1.40)*	1.10(1.08,1.12)*

1 reference category for categorical variable and * reference P-value < 0.05

The results of model four showed that the use of adequate ANC services utilization was significantly correlated with woman’s education, woman’s occupation, age, marital status, husband’s education, husband’s occupation, sex of household head, media exposure, distance to health facilities, getting the money needed for treatment, residence, age at first birth, contraceptive use, women's decision-making capacity, pregnancy wanted, birth order, wealth index, and region. Women aged 35 to 49 years were 1.36 (AOR = 1.36, 95% CI 1.32–1.41) times more likely to receive adequate ANC services than women aged 15–24 years. Secondary and above-educated women had a 2.13 (AOR = 2.13, 95% CI 2.07–2.19) times higher likelihood of receiving adequate ANC services than women without formal education. Workingwomen were 1.17 (AOR = 1.17, 95% CI 1.14–1.17) times more likely to receive adequate ANC services than housewives women. Women whose husbands had attended primary school had 1.27(AOR = 1.27, 95% CI 1.24–1.32) times higher likelihood of receiving adequate ANC services than women whose husbands had not attended formal education. Women whose husbands had attained secondary education and higher had a 1.55 (AOR = 1.55, 95% CI 1.51–1.60) times higher likelihood of receiving adequate ANC services than women whose husbands had no formal education. Rich women had 1.26 (AOR = 1.26, 95% CI 1.24–1.29) times higher odds of receiving adequate ANC services than poor women. Compared to women who were exposed to the media, those who were not exposed to it were 0.74 (AOR = 0.74, 95% CI 0.72–0.75) times less likely to receive adequate ANC services. Women with unwanted pregnancies had 0.81 (AOR = 0.81, 95% CI 0.78–0.84) times lower odds of receiving adequate ANC service compared to those with wanted pregnancies. Women with birth orders 5 and above had 0.73 (AOR = 0.73, 95% CI 0.68–0.78) times lower chance of receiving adequate ANC services than women with first birth orders. Women in rural areas had a 0.80 (AOR = 0.80; 95% CI 0.78–0.82) lower likelihood of receiving adequate ANC services than women in urban areas. Women who used contraception were 1.39 (AOR = 1.39; 95% CI 1.36–1.42) times more likely to receive adequate ANC service than women who did not use contraception. Women who had autonomy decided with their husbands had 0.83 (AOR = 0.83; 95% CI 0.81–0.86) times higher likelihood of receiving adequate ANC service than Women who had autonomy on their own. The odds of adequate ANC service were 1.13 (AOR = 1.13; 95% CI 1.11–1.16) higher among women where the distance to a health facility is a big problem compared to women whose distance to a health facility is not a big problem. Women in the western and southern regions had 1.37 times (AOR = 1.37, 95% CI 1.33–1.40) and 1.22 times (AOR = 13.42, 95% CI 1.16–1.29) higher odds of receiving adequate ANC visits than women in the central region (Table 3).

### Random effect analysis

The results of the random effect model are summarized in Table 4. The ICC value in the null model indicates that there was a 27.21% variation in pregnant women's use the adequate ANC services across clusters, which was reduced to about 12.23% after taking into account both individual and community-level variables in the full model. The final model's PCV value revealed that the combined effect of individual and community-level variables accounted for 62.60% of the variation in the community-wide prevalence of adequate ANC services utilization. Furthermore, the MOR of 2.87 in the null model indicated the presence of heterogeneity in adequate ANC services utilization between clusters. This demonstrates that pregnant women in clusters with higher adequate ANC services utilization prevalence were more than twice as likely to use it as those in clusters with lower adequate ANC services utilization rates. Model IV had the lowest deviance value (304,418.6), AIC (304,484.6), and BIC (304,827.5) because it was the best-fitting model (Table 4).

**Table 4:** Measures of variation and model fit statistics on adequate ANC services utilization in sSA countries

Measures of variation	Model I (null model)	Model II	Model III	Model IV (full model)
Variance (95% CI)	1.23 (1.03, 1.43)*	0.60 (0.49,0.71)*	0.71 (0.58,0.83)*	0.46 (0.37, 0.58)*
PCV (%)	Reference	51.22	42.28	62.60
ICC (%)	27.21	15.42	17.75	12.23
MOR	2.87	2.09	2.23	1.90
Model fit statistics				
DIC (-2log likelihood)	329,564.4	307,612.4	317,130.2	304,418.6
AIC	329,568.4	307,668.4	317,146.2	304,484.6
BIC	329,589.2	307,959.4	317,229.3	304,827.5

^*^Reference *P*-value < 0.001

## Discussion

The pooled prevalence of adequate ANC service utilization in sub-Saharan Africa (sSA) was 55% (95% CI: 54–56). It was higher than South Asian countries (46.64%) [18] and East African countries (52.44%) [19]. These disparities could be attributed to the presence of health system infrastructure, sample size, policy variations against maternal health care services, survey year, variability in awareness of maternal health care services, and socio-cultural differences between countries. The results of the random effects analysis showed that variables at the community and individual levels were responsible for approximately 62.60% of the variation in the use of adequate ANC services. Similar finding in sSA countries [38] and South Asian countries [18] revealed that community and individual-level factors contributed to a large difference in the utilization of adequate ANC services across communities. This is because women who reside in the same communities are more likely to have similar results than women who live in different communities because they share similar characteristics and have access to the same maternal health care.

The two-level logistic regression model revealed that woman’s education, woman’s occupation, age, marital status, husband’s education, husband’s occupation, sex of household head, media exposure, distance to health facilities, getting the money needed for treatment, residence, age at first birth, contraceptive use, women's decision-making capacity, pregnancy wanted, birth order, wealth index, and region were significantly associated with the use of adequate ANC service utilization.

The study found that maternal and husband education was a strong predictor of ANC visit utilization. The likelihood of receiving adequate ANC increases as the women's and husband's education levels rise. This is in line with the studies carried out in South Asian countries [18], Debre Tabor Town, northwest Ethiopia [39], South Gondar Zone, Northwest Ethiopia [14], Ethiopia [40], Northern Ghana [15], Nepal [41]and sub-Saharan Africa countries [38]. Their possible justification could be that education improves maternal healthcare service utilization and increases knowledge of specific issues. Furthermore, empowering women through education, household wealth, and decision-making improves maternal healthcare service utilization.

Women aged 35 to 49 years were more likely to receive adequate ANC services than women aged 15 to 24 years. This finding is in line with studies done in Debre Tabor Town, northwest Ethiopia [39], rural Ghana [42], Indonesia [43] Nepal [41], and the Philippines and Indonesia [44]. The possible reason for this is that older women in the higher age’s have more experienced knowledge about the importance of maternal health due to having more exposure to health care services information during pregnancy. The findings also showed that the odds of receiving adequate ANC increase as women's age at first birth increases. This finding was in agreement with a study done in South Asian countries [18]. Working women were more likely to receive adequate ANC services than housewives women. This finding is similar to a study in Nepal [41], Bangladesh [45], Indonesia [43] and sub-Saharan Africa [38]. This could be because working women benefit from a pregnancy care health insurance system and share maternal health care knowledge with coworkers. Female household heads were more likely to receive adequate ANC services than their male counterparts. This finding was supported by a study conducted in Ethiopia [46]

Women who had autonomy on their own had a higher likelihood of receiving adequate ANC than women who had autonomy decided with their husbands. This finding is supported by studies conducted in Nepal [41], South Asian countries [18], and sub-Saharan Africa [38]. This could be because women's autonomy in terms of maternal healthcare utilization allows them to make decisions about their healthcare. The study also showed that married women had a higher likelihood of receiving adequate ANC services than single (divorced/windowed) women. This result is consistent with studies conducted in rural Ghana [43]and Indonesia [43]. Compared to their unmarried counterparts, the married women's planned and desirable pregnancies, societal acceptance and support of their pregnancy state, and the psychological and financial support they received from their husbands may all have contributed to the higher ANC service adequacy [16].

The wealth index was significantly associated with adequate use of ANC services. Women with a rich wealth index were more likely to receive adequate ANC services than poor women. This result is in line with studies done in Ethiopia [40], Northern Ghana [15], South Asian countries [18], and sub-Saharan African countries [38]. This could be because rich women have more access to healthcare and receive important information about adequate ANC services through mass media. Furthermore, this may be attributed to the indirect cost of ANC, such as transport costs when traveling to distant health facilities.

Women in urban areas had a higher chance of receiving adequate ANC services than rural women. This is consistent with the studies conducted in Ethiopia [40], South Asian countries [18], South Gondar Zone, Northwest Ethiopia [14], Northern Ghana [15], Indonesia [47], Nepal [41], and sub-Saharan Africa countries [38]. The possible explanation could be that urban women may have better education, and access to maternal health services, and are more aware of the importance of receiving adequate ANC services. The odds of adequate ANC visits were higher among women where distance to a health facility is a big problem compared to women whose distance to a health facility is not a big problem. This is supported by the previous studies conducted in Delta State, the Southern part of Nigeria [48], Indonesia [47], and sub-Saharan African countries [38].

Mass media exposure is associated with adequate ANC. Women who were not exposed to media received more adequate ANC services than women who were exposed to media. This finding is supported by a study done in Ethiopia [40], Delta State, Southern part of Nigeria [48], South Asian countries [18], South Gondar Zone, Northwest Ethiopia [14], Bangladesh [45], and sub-Saharan Africa countries [38]. This is because women who received messages about maternal health care services via radio, television, and newspapers were more likely to use ANC services.

Women with unwanted pregnancies were less likely to receive adequate ANC services than those with wanted pregnancies. This finding is in line with studies done in Debre Tabor Town, northwest Ethiopia [39], South Gondar Zone, Northwest Ethiopia [14], and sub-Saharan African countries [38]. This could be attributed to unplanned pregnancies and unwillingness to seek adequate ANC services. The lack of a pregnancy mindset, which is common in unplanned pregnancies, may have negatively impacted mothers' use of ANC services. If the pregnancy is planned, women want to have a healthy pregnancy and may pay special attention to receiving adequate ANC service.

Women who used contraception were more likely to receive adequate ANC services than women who did not use contraception. This finding was in agreement with a study done in sSA countries [38]. Women with birth orders 2–4 and 5 and above had a lower chance of receiving adequate ANC services than women with first birth orders. A similar finding was also found in Ethiopia [49] and sub-Saharan African countries [38].

Geographic region was found to be significantly associated with adequate use of ANC services in sub-Saharan Africa. Women in Southern and Western sub-Saharan Africa were more likely to use ANC services than women in Central sub-Saharan Africa. This is in line with the studies carried out in sub-Saharan African countries [38]. This could be due to differences in the availability of medical facilities. Women from western sub-Saharan Africa, where the quality of health care services is better than in central sub-Saharan Africa, are more informed about maternal health care services as a result of economic and technological advances.

## Conclusion

Sub-Saharan Africa (sSA) countries had a low pooled prevalence of adequate ANC service utilization with high inequality. The finding found that there is a significant variation in the utilization of adequate ANC services among the sSA countries. Age at first birth, contraceptive use, women's decision-making capacity, desired pregnancy, birth order, wealth index, region, media exposure, occupation, age, marital status, husband's educational level, sex of the head of the household, and distance to health facilities were all significantly correlated with the use adequate ANC service utilization in sSA countries. Therefore, public health initiatives should focus on young women, rural women, poor women, and uneducated women and their husbands to enhance maternal health services utilization. Furthermore, policies and programs that address cluster variations in the utilization of adequate ANC services must be developed, and their implementation must be vigorously pursued.

## Data Availability

Data are available online and you can access it from www.measuredhs.com

## Abbreviations

AIC: Akaike’s information criterion
ANC: Antenatal care
AOR: Adjusted odds ratio
CI: Confidence intervals
DIC: Deviance information criterion
EAs: Enumeration areas
DHS: Demographic and Health Survey
ICC: Intra-cluster correlation
MOR: Median odds ratio
sSA: Sub-Saharan Africa
PCV: Proportional change in variance
PNC: Postnatal care
WHO: World Health Organization
